# Sulfur Oxidation in the Acidophilic Autotrophic *Acidithiobacillus* spp.

**DOI:** 10.3389/fmicb.2018.03290

**Published:** 2019-01-10

**Authors:** Rui Wang, Jian-Qiang Lin, Xiang-Mei Liu, Xin Pang, Cheng-Jia Zhang, Chun-Long Yang, Xue-Yan Gao, Chun-Mao Lin, Ya-Qing Li, Yang Li, Jian-Qun Lin, Lin-Xu Chen

**Affiliations:** State Key Laboratory of Microbial Technology, Shandong University, Qingdao, China

**Keywords:** *Acidithiobacillus*, sulfur oxidation, two-component system, elemental sulfur oxidation, thiosulfate oxidation pathways, sulfide oxidation, sulfite oxidation

## Abstract

Sulfur oxidation is an essential component of the earth’s sulfur cycle. *Acidithiobacillus* spp. can oxidize various reduced inorganic sulfur compounds (RISCs) with high efficiency to obtain electrons for their autotrophic growth. Strains in this genus have been widely applied in bioleaching and biological desulfurization. Diverse sulfur-metabolic pathways and corresponding regulatory systems have been discovered in these acidophilic sulfur-oxidizing bacteria. The sulfur-metabolic enzymes in *Acidithiobacillus* spp. can be categorized as elemental sulfur oxidation enzymes (sulfur dioxygenase, sulfur oxygenase reductase, and Hdr-like complex), enzymes in thiosulfate oxidation pathways (tetrathionate intermediate thiosulfate oxidation (S_4_I) pathway, the sulfur oxidizing enzyme (Sox) system and thiosulfate dehydrogenase), sulfide oxidation enzymes (sulfide:quinone oxidoreductase) and sulfite oxidation pathways/enzymes. The two-component systems (TCSs) are the typical regulation elements for periplasmic thiosulfate metabolism in these autotrophic sulfur-oxidizing bacteria. Examples are RsrS/RsrR responsible for S_4_I pathway regulation and TspS/TspR for Sox system regulation. The proposal of sulfur metabolic and regulatory models provide new insights and overall understanding of the sulfur-metabolic processes in *Acidithiobacillus* spp. The future research directions and existing barriers in the bacterial sulfur metabolism are also emphasized here and the breakthroughs in these areas will accelerate the research on the sulfur oxidation in *Acidithiobacillus* spp. and other sulfur oxidizers.

## Introduction

*Acidithiobacillus*, the gram-negative sulfur-oxidizing chemolithotrophic bacteria in the proteobacterial class *Acidithiobacillia*, formerly belonged to the genus “*Thiobacillus*” (Vishniac and Santer, 1957; Kelly and Wood, 2000; Williams and Kelly, 2013). However, due to their higher acid-tolerance and relatively closer evolutionary relationships with each other compared to other species in the genus *Thiobacillus*, they were reclassified as a new genus “*Acidithiobacillus*” in 2000 (Kelly and Wood, 2000). Members of this genus have the remarkable capability of oxidizing various reduced inorganic sulfur compounds (RISCs) to obtain electrons for carbon dioxide fixation, and some of them also have ferrous iron oxidation ability (Harrison, 1984). On the basis of physiological characters and 16S rRNA gene sequence comparisons, the genus *Acidithiobacillus* has been classified into seven different species (Table 1; Waksman and Joffe, 1922; Temple and Colmer, 1951; Hallberg and Lindstrom, 1994; Xia et al., 2007; Liljeqvist et al., 2012; Falagan and Johnson, 2016; Nunez et al., 2017). Based on the differences in the energy-substrates, species in *Acidithiobacillus* can be divided into two groups: the sulfur-oxidizing-only species, including *Acidithiobacillus thiooxidans*, *Acidithiobacillus caldus* and *Acidithiobacillus albertensis*, and the sulfur- and ferrous- oxidizing species, including *Acidithiobacillus ferrooxidans*, *Acidithiobacillus ferrivorans*, *Acidithiobacillus ferriphilus*, and *Acidithiobacillus ferridurans* (Table 1). *Acidithiobacillus* strains are widely distributed in acidic sulfur-containing environments on land or in the sea, including soil, sediments, hot springs, iron-sulfur mineral deposits and acid mine drainage (AMD), where these bacteria participate in the global element cycles of sulfur and iron, promoting the oxidation of RISCs to sulfate and the conversions between ferrous and ferric ions (London and Rittenberg, 1964; Nielsen and Beck, 1972; Taylor et al., 1984; Schrenk et al., 1998; Jones et al., 2012; Hua et al., 2015; Sharmin et al., 2016).

**Table 1:** Taxonomic traits of seven species in the genus of *Acidithiobacillus*.

Trait	*A. ferrooxidans*	*A. ferrivorans*	*A. ferriphilus*	*A. ferridurans*	*A. thiooxidans*	*A. caldus*	*A. albertensis*
Gram stain	-	-	-	-	-	-	-
Cell size (μm)	1.0 × 0.5	2.4 × 0.5	1–2	1–2	1.0–2.0 × 0.5	1.2–1.9 × 0.7	1–2 × 0.4–0.6
Motility	+/-	+	+	+	+	+	+
Growth pH (optimum)	1.3–4.5 (2.0–2.5)	1.9–3.4 (2.5)	1.5 (2.0)	1.4–3.0 (2.1)	0.5–5.5 (2.0–3.0)	1.0–3.5 (2.0–2.5)	0.5-6.0 (3.5–4.0)
Growth T/°C (optimum)	10–37 (30–35)	4–37 (28–33)	5–33 (30)	10–37 (29)	10–37 (28–30)	32–52 (40–45)	10–40 (25–30)
Oxidation of S^0^, S_4_O_6_^2-^, S_2_O_3_^2-^	+	+	+	+	+	+	+
Oxidation of Fe^2+^	+	+	+	+	-	-	-
Growth on sulfide minerals	+	+	+	+	-	-	-
Growth on hydrogen	+	(+)	-	+	-	+	NR
Anaerobic growth with Fe^3+^	+	+	+	+	-	-	-
N_2_ fixation	+	+	NR	NR	-	-	-
Mol% G+C	58–59	55–56	57.4	58.4	52	63–64	61.5
Thiosulfate-metabolic pathways	TSD enzyme; S_4_I pathway	Sox system; TSD enzyme; S_4_I pathway	NR	TSD enzyme; S_4_I pathway	Sox system; S_4_I pathway	Sox system; S_4_I pathway	Sox system; S_4_I pathway
Reference	Janiczek et al., 2007; Valdés et al., 2008a,b; Hallberg et al., 2010; Hedrich and Johnson, 2013b; Kikumoto et al., 2013	Hallberg et al., 2010; Hedrich and Johnson, 2013b; Christel et al., 2016	Falagan and Johnson, 2016	Hedrich and Johnson, 2013a,b; Miyauchi et al., 2018	Valdés et al., 2008b; Hallberg et al., 2010; Yin et al., 2014	Valdés et al., 2008b; Hallberg et al., 2010; Mangold et al., 2011; Hedrich and Johnson, 2013b	Bryant et al., 1983; Xia et al., 2007; Castro et al., 2017


*+, positive; -, negative; +/-, the positive or negative result from different reports; (+), some strains have the ability to oxidize hydrogen; NR, not reported; Tm, temperature.*

*Acidithiobacillus* spp. are prevalent in acid mines due to their capabilities of utilizing the sulfur and iron in ores and adapting to extremely acidic environments. As a consequence, *Acidithiobacillus* spp. have become the most active bacteria used in the biohydrometallurgy industry in bioleaching or biomining, whereby metals are extracted from ores through microbial oxidation (Rawlings et al., 1999; Rawlings, 2005). Three species, *A. ferrooxidans A. thiooxidans*, and *A. caldus*, have been studied extensively and applied widely in bioleaching for mineral extraction from ores (Valdés et al., 2008b). In addition, the ability of heavy metal leaching has expanded the application of *Acidithiobacillus* spp. from hydrometallurgy to the treatment of wastes containing heavy metals, such as sewage sludge, spent household batteries, mine tailings, and printed circuit boards (Pathak et al., 2009; Bayat and Sari, 2010; Arshadi and Mousavi, 2014; Ijadi Bajestani et al., 2014; Nguyen et al., 2015; Rastegar et al., 2015). Moreover, these bacteria have been widely studied in microbial desulfurization of coal and gas (Azizan et al., 2000; He et al., 2012; Charnnok et al., 2013). Taken together, *Acidithiobacillus* spp. have shown their great value of applications not only in metal leaching (copper, uranium, gold and so on) from mineral ores, but also in solving environmental pollution problems caused by heavy metals and inorganic sulfur compounds.

Sulfur oxidation, as the essential physiological feature of *Acidithiobacillus* spp. and the important character for their application, has attracted extensive attention (Suzuki and Werkman, 1959; London, 1963; London and Rittenberg, 1964; Suzuki et al., 1992; Hallberg et al., 1996; Quatrini et al., 2009; Chen et al., 2012; Yin et al., 2014; Wang et al., 2016; Nunez et al., 2017; Zhang et al., 2018). The element sulfur can exist in various oxidation states ranging from -2 to +6, which results in a variety of RISCs including tetrathionate (S_4_O_6_^2-^), thiosulfate (S_2_O_3_^2-^), sulfite (SO_3_^2-^), sulfide (S^2-^), and elemental sulfur (S^0^). A variety of enzymes and proteins involved in the oxidation of RISCs were discovered, including sulfur-oxidizing enzymes, sulfur transferases and sulfur carrier proteins. The sulfur-metabolic enzymes in *Acidithiobacillus* spp., based on their substrates, can be categorized as elemental sulfur oxidation enzymes, enzymes in thiosulfate oxidation pathways, sulfide oxidation enzymes, and sulfite oxidation enzymes. These enzymes work cooperatively to oxidize the RISCs to the final product sulfate. Therefore, the identification of novel sulfur-metabolic proteins and investigation of the metabolic and regulatory mechanisms of these known sulfur oxidation proteins have been the main subject of researches on sulfur oxidation in *Acidithiobacillus* spp. Significant research progress was made over the last decades in these areas. Here, the key points are summarized to provide an overall picture of sulfur oxidation in *Acidithiobacillus* spp.

## Elemental Sulfur Oxidation

### Sulfur Dioxygenase (SDO)

Elemental sulfur (S^0^), mainly present in the form of insoluble homocyclic S_8_, is hydrophobic, metastable, and almost insoluble in water. Elemental sulfur oxidation activity was first detected in *A. thiooxidans* as early as 1959 (Suzuki and Werkman, 1959). The purified enzyme that is associated with this activity was named sulfur dioxygenase (SDO, EC 1.13.11.18) and reduced glutathione (GSH) was necessary for activity detection in *in vitro* assays. It was reported that Sulfur dioxygenase (SDO) is composed of a 21- and a 26- kDa protein in *A. thiooxidans* or two 23 kDa subunits in *A. ferrooxidans* (Suzuki, 1965; Silver and Lundgren, 1968a; Sugio et al., 1987a). Assays *in vitro* revealed that the actual substrate for SDO-catalyzed reaction was the sulfane sulfur atom of glutathione persulfide (GSSH) and its homologs (GSSnH, *n* > 1) (Rohwerder and Sand, 2003, 2008), but the amino acid sequences of SDO were not identified at that time.

In recent years, SDO activity was also detected in the mitochondria of plants and animals and in heterotrophic bacteria, and protein sequences were identified in these organisms. This enzyme was originally named as SDO in *Acidithiobacillus* spp. Based on the further studies, two new names, ETHE1 and persulfide dioxygenase (PDO), were proposed for SDO homologs in mitochondria and heterotrophic bacteria, respectively (Jackson et al., 2012; Kabil and Banerjee, 2012; Liu H. et al., 2014; Sattler et al., 2015). To avoid any confusion, here we use SDO throughout this paper to refer to this enzyme in *Acidithiobacillus* spp. The mitochondrial ETHE1s identified in human and *Arabidopsis* both belong to the metallo-β-lactamase superfamily, and their metal-binding active sites consist of an aspartate and two histidine residues (Tiranti et al., 2009; Holdorf et al., 2012). Based on phylogenetic analysis, heterotrophic bacterial PDOs were classified into three subgroups (Liu H. et al., 2014). Structural analyses indicated that the PDOs in heterotrophic bacteria contained the conserved amino acid residues, and differences in the GS-moiety binding sites of the key amino acid residues supported grouping of PDOs (Sattler et al., 2015). The major physiological function of SDOs in mitochondria or in heterotrophic bacteria has been proposed: ETHE1s/ PDOs works cooperatively with sulfide:quinone oxidoreductase (SQR) to oxidize H_2_S, relieving the toxic effect of H_2_S to the cells (Guimaraes et al., 2011; Kabil and Banerjee, 2012; Liu H. et al., 2014).

Sulfur dioxygenase in *Acidithiobacillus* spp. was previously proposed to serve as the first enzyme for extracellular elemental sulfur oxidation. This was concluded from a SDO-dependent sulfur oxidation model: extracellular elemental sulfur was first activated by thiol-containing outer-membrane proteins to generate persulfide sulfane sulfur, and this product was further oxidized by periplasmic SDO to produce sulfite (Rohwerder and Sand, 2003). The nucleotide and amino acid sequences of SDO in *Acidithiobacillus* spp. were not identified until 2014 (Wang et al., 2014). Two homologs of human ETHE1, AFE_0269 in *A. ferrooxidans* ATCC 23270 and A5904_0790 in *A. caldus* MTH-04, exhibited a remarkable GSH-dependent SDO activity in *in vitro* assays (Wang et al., 2014). Recently, a second SDO (A5904_0421, termed SDO1), with 33% amino acid identity to previously identified Ac-SDO (A5904_0790, termed SDO2), was identified in *A. caldus* MTH-04. The enzymatic activity of SDO1 is much lower than that of SDO2 (Wu et al., 2017). The latest study on ETHE1-like SDO (SDO2 homolog) from *A. caldus* C-SH12 suggested that AcSDO is a homotetramer containing a mononuclear iron site with a 2-His-1-carboxylate facial triad in the active site. The key amino acid residues of this protein were described recently (Ruhl et al., 2018).

**FIGURE 1:**
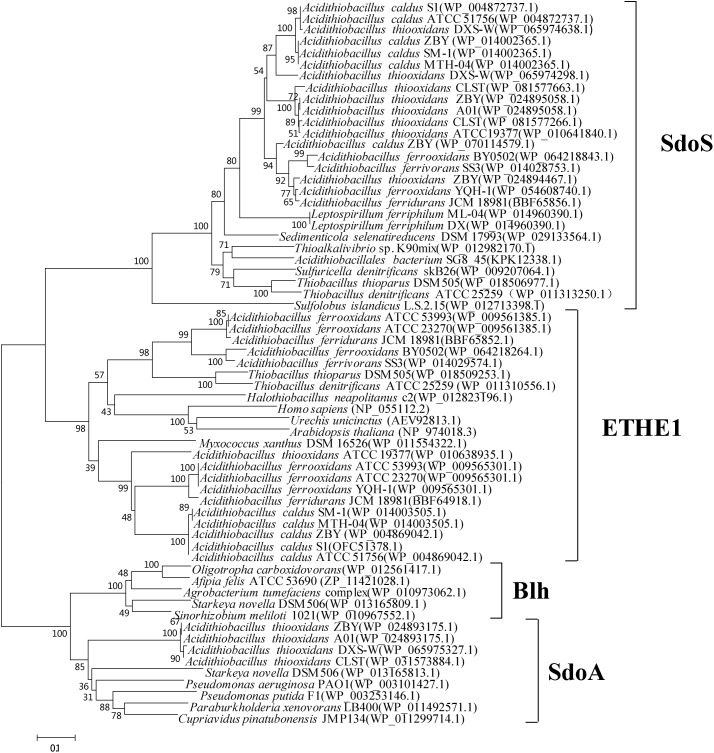
Phylogenetic analysis of SDOs in *Acidithiobacillus* spp. and other typical prokaryotes. MEGA 5.0 with the bootstrap test (1000 replicates) were used to construct NJ-tree. The protein ID or locus_tag of each SDO is present in parentheses. The predicted functional domains of proteins whose sequences identities are higher than 30% are selected for this phylogenetic analysis.

There are two to three copies of SDO paralogs in different species of *Acidithiobacillus* (Figure 1; Wu et al., 2017). *A. caldus* SDO1 and its homologs form a new subgroup distinct from the well described subgroups (ETHE1, Blh, SdoA), named as SdoS, whereas the SDO2 of *A. caldus* and its homologs belong to the ETHE1 subgroup (Figure 1; Wu et al., 2017). Phylogenetic analysis also revealed that some potential SDOs from *A. thiooxidans* belong to the SdoA subgroup (Figure 1). Studies on the SDOs indicated the ubiquitous existence of this elemental sulfur-oxidizing enzyme in these chemoautotrophic sulfur-oxidizing bacteria.

Recently, further phylogenetic analysis on bacterial PDOs suggested PDOs can be reclassified into three types (Xia et al., 2017). The subgroups of SdoA and Blh were categorized into the type II PDOs, and ETHE1 homologous proteins are grouped as type I PDOs. The type III PDOs exhibited low sequence identities with the type I and type II PDOs. According to the new classification of PDOs, the SDOs in the SdoS subgroup belong to the type III category. Studies on SDOs in SdoS and ETHE1 subgroups found that there are obvious differences in the key amino acid residues of the substrate binding regions (unpublished data), indicating different functions of SDO1 and SDO2 in the process of sulfur oxidation in *Acidithiobacillus* spp.

The role of SDOs in sulfur oxidation in *Acidithiobacillus* was studied using *sdo* deletion and overexpression strains. When grown in liquid S^0^-medium, the *sdo* (AFE_0269) mutant of *A. ferrooxidans* ATCC 23270 grew much slower than the wild type. Moreover, cell extracts of the mutant still maintained the SDO activity when cultivated in S^0^- or Fe^2+^- medium (Wang et al., 2014). No significant differences in growth were observed among the three *sdo* mutants of *A. caldus* MTH-04 (Δ*sdo*1, Δ*sdo*2, and Δ*sdo*1&2) and the wild type strain in S^0^-media, and the SDO activities of cell extracts from these mutants were not lower than that from wild type (Wu et al., 2017). All these results indicated that the absence of SDOs in *A. ferrooxidans* and *A. caldus* neither impaired their elemental sulfur oxidation activities nor caused lethal effects on their growth rates in S^0^-media. It was suggested that SDO1 in *A. caldus* is involved in the S_4_O_6_^2-^ metabolic process, because when cultivated in tetrathionate at a concentration of 2.27 g/L, strains Δ*sdo*1 and Δ*sdo*1&2 did not exhibit any OD increase in growth experiments (Wu et al., 2017). Transcriptional analysis on *sdo* deletion and overexpression strains of *A. caldus* showed that the transcription levels of *sdo*1 and *sdo2* had close linkages to those of *tetH* (encoding a tetrathionate hydrolase) and *sqr* (encoding a sulfide:quinone oxidoreductase), respectively (Wu et al., 2017). In contrary to the previous hypothesis that SDO functioned in the oxidation of persulfide sulfane sulfur in periplasm, it is now believed that all the SDO homologs in *Acidithiobacillus* spp. are cytoplasmic proteins due to the lack of signal peptides and transmembrane regions (Wu et al., 2017). Therefore, based on the current knowledge, SDOs in *Acidithiobacillus* spp. are believed to be involved in cytoplasmic elemental sulfur oxidation, and different subgroups of SDOs are probably responsible for the oxidation of elemental sulfur generated by different pathways: the ETHE1-subgroup of SDOs are involved in the H_2_S-oxidation pathway and the SdoS-subgroup of SDOs are related to the S_4_O_6_^2-^-decomposition pathway (Wu et al., 2017).

Sulfur oxygenase reductase (SOR) is another elemental sulfur oxidizing enzyme found in *Acidithiobacillus* spp. This enzyme, first reported in several acidophilic and thermophilic archaea, can catalyze the disproportionation of cytoplasmic elemental sulfur and generate thiosulfate, sulfite, and sulfide (Kletzin, 1989; Kletzin et al., 2004; Ghosh and Dam, 2009). The reaction is dioxygen (O_2_)-dependent with no external cofactors or electron donors required, and the oxidation is coupled with neither electron transfer nor substrate-level phosphorylation (Kletzin, 1989; Urich et al., 2004). The SORs from archaeal and bacterial species are large hollow spheres consisting of 24 identical subunits. Each SOR monomer has a catalytic pocket containing an indispensable cysteine and a low-potential non-heme iron site (Urich et al., 2006; Li et al., 2008).

**FIGURE 2:**
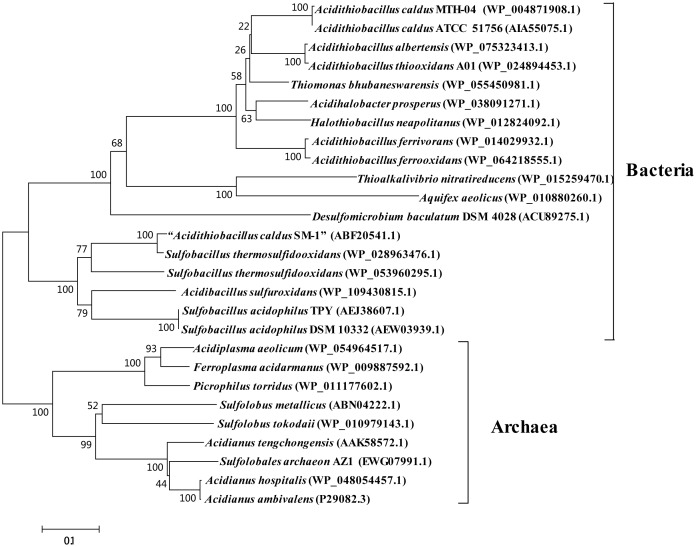
Phylogenetic analysis of SORs in some *Acidithiobacillus* spp. and other typical prokaryotes. MEGA 5.0 with the bootstrap test (1000 replicates) were used to construct NJ-tree. The protein ID or locus_tag of each SOR is present in parentheses. The predicted functional domains of proteins whose sequences identities are higher than 30% are selected for this phylogenetic analysis. The SOR reported from *Acidithiobacillus caldus* SM-1 was actually SOR from *Sulfobacillus* spp. Thus, it was marked as “*A. caldus* SM-1” in the phylogenetic tree (Chen et al., 2007; Janosch et al., 2015).

**FIGURE 3:**
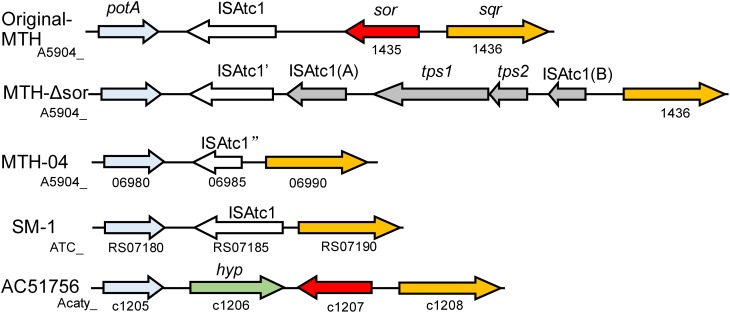
The loci of the *sor* gene on the genomes of different *A. caldus* strains. Original-MTH, *A. caldus* MTH-04 wild type harboring *sor* gene (the *sor-sqr* gene cluster sequence GenBank: MK165447); MTH-Δsor, *A. caldus* MTH-04 *sor* mutant generated by electroporation of the suicide plasmid (the *sqr* gene cluster sequence GenBank: MK165449); MTH-04, *A. caldus* MTH-04 wild type lacking *sor* gene (GenBank: CP026328.1); SM-1, *A. caldus* SM-1 (GenBank: CP002573.1); ACA, *A. caldus* ATCC 51765 (GenBank:CP005986.1); *potA*, ABC transporter ATP-binding protein; ISAtc1, IS elements; *tps1&2*, transposase; *sor*, sulfur oxygenase reductase; *sqr*, sulfide:quinone oxidoreductase; *hyp*, hypothetical protein. The gene locus on the chromosome was shown below the corresponding gene.

Although SOR activity from *A. caldus* SM-1 cultivated in bioreactors treating gold-bearing concentrates was reported (Chen et al., 2007), a subsequent study indicated that the *sor* gene was absent in the complete genome sequence of this strain (You et al., 2011). Sequence analysis of SOR proteins indicated that the SOR ascribed to *A. caldus* SM-1, was actually isolated from *Sulfobacillus* contaminated sample (Figure 2; Janosch et al., 2015). The SOR homolog was found in our laboratorial strain *A. caldus* MTH-04, and the overexpression of *sor* in *A. caldus* MTH-04 increased SOR activity by 22.2% and meanwhile resulted in a growth advantage after the mid-log phase (unpublished data). SOR was defined as a cytoplasmic S^0^-oxidizing enzyme based on studies on a *sor* deficient mutant of *A. caldus* MTH-04 (Chen et al., 2012). SOR homologs have been found in some, but not all, strains of *A. thiooxidans*, *A. ferrooxidans*, *A. ferrivorans*, *A. albertensis* (Figure 2; Valdés et al., 2008b; Yin et al., 2014; Christel et al., 2016). Phylogenetic analysis indicated that the SORs in these *Acidithiobacillus* spp. strains were probably acquired from sulfur-oxidizing archaea via horizontal gene transfer (Figure 2). Moreover, the *sor* gene could be eliminated via transposition in strain MTH-04 of *A. caldus* (Figure 3; Chen et al., 2012). For our laboratory strain MTH-04, both the electrotransformation of suicide plasmid specific for *sor* gene mutagenesis and the continuous passage in S^0^-media in the laboratory cultivation environment resulted in the loss of *sor* gene (Figure 3). The tendency of eliminating the *sor* gene in *A. caldus*, the sporadic distribution and the relatively low retention rate of the *sor* gene in *Acidithiobacillus* spp. indicate that SOR is supplementary (You et al., 2011; Chen et al., 2012; Janosch et al., 2015; Christel et al., 2016), but not necessary, for cytoplasmic elemental sulfur oxidation in these sulfur-oxidizing bacteria.

### Heterodisulfide Reductase (Hdr)-Like System

A Hdr-like complex is proposed to serve as an elemental sulfur oxidation enzyme in the cytoplasmic space of *Acidithiobacillus* and many other sulfur-oxidizing bacteria and archaea (Quatrini et al., 2009; Mangold et al., 2011; Chen et al., 2012; Inskeep et al., 2013; Yin et al., 2014; Liu L.J. et al., 2014; Dahl, 2015; Christel et al., 2016). Proteins of the Hdr-like system from bacterial and archaeal sulfur oxidizers are homologous to those in the HdrABC complex from methanogenic archaea, sulfate-reducing archaea and sulfate-reducing bacteria (Hedderich et al., 2005). HdrABC is a unique disulfide reductase that catalyzes the reversible reduction of the disulfide bond X-S-S-X coupled with the energy conservation (Thauer et al., 2008; Kaster et al., 2011; Wagner et al., 2017). Studies have shown that the HdrABC complex is composed of three subunits: HdrA carrying a typical FAD binding motif and four binding motifs for [4Fe-4S] clusters, HdrB harboring two identical non-cubane [4Fe-4S] clusters and both of these clusters are consisted of [3Fe-4S] and [2Fe-2S] subclusters, and the ferredoxin-like HdrC containing two binding motifs for [4Fe-4S] clusters (Hamann et al., 2007; Wagner et al., 2017). In methanogenic and sulfate-reducing archaea, HdrA receives the electrons from a hydrogenase and transfers them through HdrC to the heterodisulfide reductase catalytic site on HdrB (Mander et al., 2004). Unlike the HdrABC complex, the Hdr-like system in sulfur-oxidizing bacteria and archaea is encoded by a *hdrC1B1A-hyp-hdrC2B2* gene cluster and consists of at least five subunits (HdrA, HdrB1, HdrB2, HdrC1, and HdrC2) (Boughanemi et al., 2016).

Hdr-like systems have been discovered in different species of *Acidithiobacillus*, and the function of this complex was proposed to be oxidation of disulfide intermediates (most likely sulfane sulfur as in GSSH or other sulfur carriers) to sulfite in the cytoplasm (Quatrini et al., 2009; Mangold et al., 2011; Chen et al., 2012; Yin et al., 2014; Christel et al., 2016; Koch and Dahl, 2018.) The transcriptional levels of *hdr* genes were upregulated when *A. ferrooxidans* or *A. thiooxidans* were cultivated in sulfur-containing media (Ehrenfeld et al., 2013). HdrC from *A. ferrooxidans* was heterologously expressed and the recombinant protein harbored [4Fe–4S] clusters (Ossa Henao et al., 2011). However, biochemical evidence confirming the function of Hdr-like systems is still absent. Recently, an indirect genetic study indicated that a Hdr-like complex functions in the oxidation of thiosulfate to sulfite in *Hyphomicrobium denitrificans* (Koch and Dahl, 2018). Moreover, a lipoate-binding protein (LbpA) was identified as a necessary component for the Hdr-like sulfur-oxidizing system in *H. denitrificans* (Cao et al., 2018). Thus, a sulfur oxidation pathway was proposed in which the Hdr-like complex oxidizes the sulfane sulfur delivered by the sulfur carrier TusA to sulfite, and the released electrons in the reaction might be transferred via LbpA to generate NADH (Cao et al., 2018). The confirmation of the sulfur-oxidizing ability of Hdr-like complex and the sulfur-metabolizing process catalyzed by this system in *H. denitrificans*, would undoubtedly provide new insights in the elemental sulfur oxidation in the cytoplasm of *Acidithiobacillus* spp., and promote research on the Hdr-like complex in these sulfur-oxidizers.

The presence of SDO, SOR, and Hdr-like complex in *Acidithiobacillus* spp. indicates the diversity and complexity of elemental sulfur oxidation in these acidophilic bacteria. The triple *sor-sdo1-sdo2* mutant of *A. caldus* MTH-04 exhibited an increased elemental sulfur oxidation activity, indicating the existence of undetermined elemental sulfur oxidation enzymes in *Acidithiobacillus* spp. (Wu et al., 2017). Direct and conclusive evidences are needed to confirm the function of Hdr-like complex in these *Acidithiobacillus* strains. In addition, the distinct roles, the potential cooperative effects and regulation modes of the three cytoplasmic elemental sulfur oxidation enzymes in *Acidithiobacillus* spp. could be the emphasis of future researches.

### Sulfur Trafficking in *Acidithiobacillus*

The sulfur carrier proteins TusA and DsrE exist in many sulfur oxidation bacteria and archaea (Liu L.J. et al., 2014; Dahl, 2015). The *rhd-tusA-dsrE* genes were reported in *A. caldus* and *A. ferrooxidans*, and these genes, together with the Hdr-like system gene cluster (*hdrC1B1A-hyp-hdrC2B2*) and sulfite-oxidation-enzyme genes (*soeABC*), exist in one gene cluster in *A. caldus* SM-1 (*soeABC-mogA-rhd-tusA-dsrE-hdrC1-hdrB1-hdrA-hyp-hdrC2- hdrB2*, Atc_2359-2347) (Liu L.J. et al., 2014). Similar gene clusters can also be found in other species of *Acidithiobacillus* (unpublished data). The unique gene arrangement character suggests potential functional connections among elemental sulfur oxidation (Hdr-like complex), Rhd-DsrE-TusA-mediated sulfur transfer and sulfite oxidation in the cytoplasm of *Acidithiobacillus* spp. Studies on the sulfur-trafficking proteins from *Metallosphaera cuprina* and *Allochromatium vinosum* indicated that inorganic sulfur compounds were successively transferred by Rhd, DsrE, and TusA to form sulfane sulfur at the cysteine of TusA (Liu L.J. et al., 2014; Stockdreher et al., 2014; Dahl, 2015). TusA serves as a central component of cytoplasmic sulfur trafficking in sulfur-oxidizing prokaryotes (Dahl, 2015), and might deliver the sulfane sulfur to the Hdr-like sulfur-oxidizing system (Cao et al., 2018). Thus, TusA can be considered as a joint between sulfur trafficking mediated by Rhd/DsrE/TusA and the sulfur oxidation catalyzed by Hdr-like complex in these sulfur-oxidizers. The discovery of conserved genetic clusters in several *Acidithiobacillus* species suggested that similar sulfur trafficking and oxidation pathways might also work in the cytoplasm of these sulfur-oxidizers. Thus, it would be interesting to perform further protein characterization assays *in vitro* and gene function studies *in vivo* to confirm the function of these proteins in the process of sulfur trafficking and oxidation in *Acidithiobacillus* spp.

Rhodanese (Rhd) might be an important functional enzyme in sulfur trafficking and sulfur oxidation of *Acidithiobacillus* (Quatrini et al., 2009; Chen et al., 2012; Liu L.J. et al., 2014; Yin et al., 2014; Koch and Dahl, 2018). Rhd belongs to the sulfurtransferase family, which is found in organisms from all three domains of life and involved in various cellular processes (Smit and Urbanska, 1986; Aminlari and Gilanpour, 1991; Cipollone et al., 2007). This enzyme is a thiosulfate:cyanide sulfur transferase (TST), which cleaves the S–S bond present in thiosulfate, producing sulfur and sulfite. Rhd activities were detected in crude enzyme extracts of *A. ferrooxidans, A. thiooxidans* and *A. caldus* (Tabita et al., 1969; Gardner and Rawlings, 2000). Genomic sequences revealed that there are multiple copies of putative *rhd* genes in the genomes of *Acidithiobacillus* (Valdés et al., 2008a, 2011; Liljeqvist et al., 2011; Yin et al., 2014). A 21-kDa rhodanese-like protein (P21) of *A. ferrooxidans* was induced when cells were grown on metal sulfides and different sulfur compounds, but the purified recombinant P21 protein did not show Rhd activity *in vitro* (Ramírez et al., 2002). Furtherly, eight rhodanese-like proteins from *A. ferrooxidans* were cloned and expressed in *E. coli*. Some of the recombinant proteins had the Rhd activities, and the others including the P21 did not show this activity, indicating the potential different physiological roles of these rhodanese-like proteins in this bacterium (Acosta et al., 2005). Rhd probably plays an essential role in sulfur oxidation in *A. caldus* since we had no success in the deletion of the *rhd* gene in the *hdr-rhd-tusA-dsrE* gene cluster (Zhang et al., 2014). Further bioinformatics analysis of these Rhd proteins is needed to reveal the sequence similarities and potential protein localizations in these autotrophic sulfur-oxidizing bacteria.

## Thiosulfate Oxidation

Thiosulfate (S_2_O_3_^2-^) plays an important role in the biogeochemical sulfur cycle. It is a common substrate and a key sulfur-metabolic intermediate oxidized by almost all sulfur-oxidizing microorganisms, thus thiosulfate metabolism is essential for these sulfur-oxidizers (Dahl and Prange, 2006; Ghosh and Dam, 2009). In acidic environments (pH < 4.0), thiosulfate can decompose to sulfur and sulfite chemically (Johnston and McAmish, 1973), and the generated sulfur exists as colloidal sulfur that cannot be used by *Acidithiobacillus* spp. To efficiently metabolize thiosulfate, all the species in the genus *Acidithiobacillus* possess complicated thiosulfate-oxidizing multi-enzyme systems to immediately oxidize thiosulfate and sulfur-transfer enzymes to transform the thiosulfate to other forms of sulfur-substrates (Valdés et al., 2008b).

### S_4_I Pathway

**FIGURE 4:**
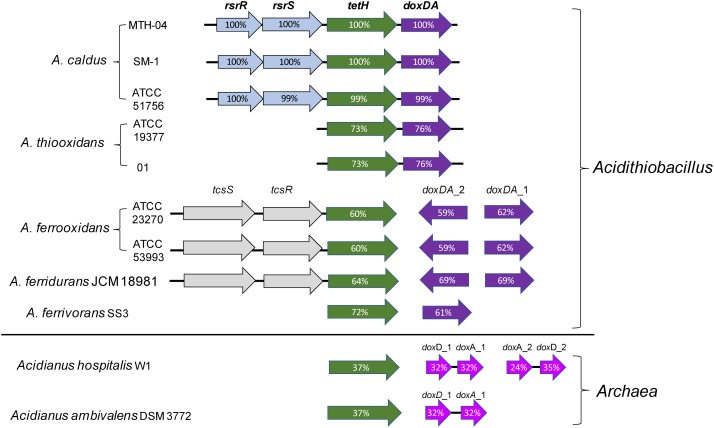
Arrangement of genes of S_4_I pathways in *Acidithiobacillus* spp. and sulfur-oxidizing archaea. The percentages of similarities between protein sequences are indicated by the values marked in the gene. Accession numbers (GenBank) for these proteins are: *A. caldus* MTH-04, RsrR (ANJ65973.1), RsrS (ANJ65974.1), TetH (OAN03451.1), DoxDA (OAN03452.1) (GenBank: MK165448); *A. caldus* SM-1, RsrR (AEK59530.1), RsrS (AEK58242.1), TetH (AEK58243.1), DoxDA (AEK58244.1); *A. caldus* ATCC 51756, RsrR (ABP38227.1), RsrS (ABP38226.1), TetH (ABP38225.1), DoxDA (ABP38224.1); *Acidithiobacillus ferrooxidans* ATCC 23270, TcsS (ACK79489.1), TcsR (ACK79259.1), TetH (ACK80599.1), DoxDA_2 (ACK79881.1), DoxDA_1 (ACK78481.1); *A. ferrooxidans* ATCC 53993, TcsS (ACH82290.1), TscR (ACH82291.1), TetH (ACH82292.1), DoxDA_2(ACH82311.1), DoxDA_1(ACH82307.1); *A.thiooxidans* ATCC 19377, TetH (WP_029316048.1), DoxDA (WP_010638552.1); *A.thiooxidans* A01, TetH (WP_024894935.1), DoxDA (WP_024894934.1); *Acidithiobacillus ferridurans* JCM 18981, TcsS (BBF65177.1), TcsR (BBF65176.1), TetH (BBF65175.1),DoxDA_2 (BBF65156.1),DoxDA_1 (BBF65160.1); *Acidithiobacillus ferrivorans* SS3, TetH (AEM46280.1), DoxDA (AEM47534.1); *Acidianus hospitalis* W1, TetH (AEE94548.1), DoxD_1 (AEE93006.1), DoxA_1 (AEE93005.1), DoxA_2 (AEE93131.1), DoxD_2 (AEE93130.1); *Acidianus ambivalens* DSM 3772, TetH (CBY66038.1), DoxD (CAA69986.1), DoxA(CAA69987.1).

A thiosulfate oxidation pathway via formation of tetrathionate as an intermediate (S_4_I) is widely found in β- and γ- proteobacteria, particularly in obligately chemolithotrophic genera including *Acidithiobacillus*, *Thermithiobacillus*, *Halothiobacillus*, and *Tetrathiobacter* (Dam et al., 2007; Ghosh and Dam, 2009). The S_4_I pathway is made up of a thiosulfate:quinol oxidoreductase (TQO or DoxDA) and a tetrathionate hydrolase (TetH or TTH). TQO oxidizes thiosulfate to tetrathionate while TetH hydrolyzes tetrathionate to thiosulfate and other products. The fact that the predicted *tetH* and *doxDA* genes are found in all the published genomes of *Acidithiobacillus* (Table 1), indicates the universality and importance of the S_4_I pathway in these acidophilic chemotrophic bacteria (Rzhepishevska et al., 2007; Wang et al., 2016). As shown in Figure 4, *tetH* and *doxDA* are arranged in a cluster in *A. caldus* and *A. thiooxidans*, while they are located separately in the genomes of *A. ferrooxidans*, *A. ferrivorans*, and *A. ferridurans*. Two copies of *doxDA* genes are located separately in the genomes of *A. ferrooxidans* and *A. ferridurans*. There are two-component systems (TCSs) located upstream of the *tetH* genes in *A. caldus*, *A. ferrooxidans*, and *A. ferridurans*. Moreover, the transcription of *tetH* and *doxDA* is influenced significantly by different sulfur-substrates in the media (Mangold et al., 2011; Chen et al., 2012; Yin et al., 2014; Christel et al., 2016), indicating that *Acidithiobacillus* spp. can modulate the S_4_I pathway at the transcriptional level in response to the various sulfur-metabolites in the growth environment.

Thiosulfate:quinol oxidoreductase (TQO), first discovered in *Acidianus ambivalens* (*A. ambivalens*), is composed of two 28-kDa DoxA and two 16-kDa DoxD subunits that formed a α_2_β_2_-tetramer (Muller et al., 2004). The membrane-bound TQO oxidizes thiosulfate to tetrathionate, and ferricyanide or decylubiquinone (DQ) takes the electrons generated from this reaction to the electron transport chain (Muller et al., 2004). Phylogenetic analysis indicated that the subunits DoxD and DoxA are fused into one protein in *Acidithiobacillus* spp. (Wang et al., 2016; Figure 4), indicating differences of TQOs between archaeal and bacterial species. Until now, the catalytic mechanism of TQO in *Acidithiobacillus* spp. is still unclear, and the functional role of TQO in sulfur oxidation needs to be further confirmed experimentally.

The hydrolysis of tetrathionate by tetrathionate hydrolase has been studied extensively in *Acidithiobacillus*, including enzymatic properties, protein localization and function in the sulfur-metabolic network (Ralf et al., 1987; de Jong et al., 1997; Bugaytsova and Lindstrom, 2004; Kanao et al., 2010, 2018; Beard et al., 2011; Yu et al., 2014). The fact that Δ*tetH* strains of *A. ferrooxidans* and *A. caldus* could not survive in tetrathionate-medium but could grow on other sulfur-substrates, indicates that tetrathionate-metabolism in these sulfur-oxidizing bacteria is TetH-dependent (van Zyl et al., 2008; Yu et al., 2014). In addition to its role in tetrathionate hydrolysis, a ferric reductase activity associated with TetH was reported for *A. ferrooxidans* as well (Sugio et al., 2009).

TetH proteins purified from *A. thiooxidans*, *A. ferrooxidans*, and *A. caldus* are all homodimers and the optimal activities are detected under acidic conditions (pH 3.0–4.0) (Hazeu et al., 1988; Sugio et al., 1996; Tano et al., 1996; Bugaytsova and Lindstrom, 2004; Kanao et al., 2007, 2018; Beard et al., 2011). Studies on the products of tetrathionate hydrolysis catalyzed by TetH proteins from different species of *Acidithiobacillus* yielded conflicting results. When suspensions of *A. ferrooxidans* were used in the assays, polythionates (up to S_13_O_6_^2-^) and sulfur rings (S^0^, containing 98% S_8_, and small amounts of S_6_, S_7_, S_9,_ and S_12_) were detected in tetrathionate hydrolysates (Ralf et al., 1987). However, using the pure enzyme from *A. ferrooxidans*, the end products were thiosulfate, sulfur and sulfate (de Jong et al., 1997). TetH assays for *A. thiooxidans* showed that thiosulfate was a main product of tetrathionate decomposition and the thiosulfate probably was further decomposed to generate elemental sulfur (Tano et al., 1996). In contrast, for *A. caldus*, thiosulfate and pentathionate were detected after TetH assays (Bugaytsova and Lindstrom, 2004). These different results are probably due to the differences in the detection methods or the protein components used in the assays. It is possible that the final products of TetH-mediated reactions in *Acidithiobacillus* spp. are thiosulfate, sulfur and sulfate, even though the intermediate metabolites may exist.

Meanwhile, the catalytic mechanism of TetH is still unclear. Crystallization and preliminary X-ray diffraction analysis of TetH from *A. ferrooxidans* was published (Kanao et al., 2013). The crystal of recombinant Af-TetH was a hexagonal cylinder with dimensions of 0.2 mm × 0.05 mm × 0.05 mm, and the crystal diffracted to 2.15 Å resolution. However, the three-dimensional structure was not obtained until now. It was reported that the sole cysteine residue (Cys301) in Af-TetH was involved in neither the tetrathionate hydrolysis reaction nor the subunit assembly, indicating a novel cysteine-independent reaction mechanism for this enzyme (Kanao et al., 2014).

Different speculations on the localization of TetH in *Acidithiobacillus* spp. were made based on the results of indirect biochemical assays. Both the low optimal pH (∼4) for TetH activities and the purification of TetH proteins from the soluble fraction implied that TetH proteins from *A. ferrooxidans* and *A. thiooxidans* were periplasmic proteins (Tano et al., 1996; de Jong et al., 1997; Kanao et al., 2007). The TetH activity analysis on different fractionations of *A. caldus* cells indicated TetH was a potential periplasmic protein (Bugaytsova and Lindstrom, 2004). However, the requirement of an acidic environment in the *in vitro* refolding experiments on recombinant Af-TetH indicated TetH probably localized in the outer membrane of *A. ferrooxidans* (Kanao et al., 2010). Results based on marine *A. thiooxidans* strain SH also suggested that TetH locates on the outer membrane of the cell (Kanao et al., 2018). Another research group proposed that TetH in *A. ferrooxidans* was probably secreted to the extracellular space (Beard et al., 2011). Signal peptides of TetHs from different strains of *Acidithiobacillus* spp. were predicted in bioinformatics analysis (unpublished data), implying TetH was secreted into the periplasm of these sulfur-oxidizing bacteria. Further immunocytochemical experiments and micro-imaging technologies are required to obtain conclusive evidence for the localization of this enzyme.

### Sox System

The sulfur oxidizing enzyme (Sox) system, a typical periplasmic multi-enzyme system, was first found in lithoautotrophic *Paracoccus pantotrophus* (Friedrich et al., 2000, 2005). This complex has been thought to be widely distributed among the various phylogenetic groups of photo- and chemo-lithotrophic sulfur-oxidizing prokaryotes (summarized by Ghosh and Dam, 2009; Frigaard and Dahl, 2009). The Sox system in *P. pantotrophus* is made up of four components: SoxXA, SoxYZ, SoxB, and Sox(CD)_2_ (Friedrich et al., 2005). This typical Sox system has the capability of oxidizing thiosulfate, sulfide, sulfite, and elemental sulfur to sulfate as the final product (summarized by Ghosh and Dam, 2009). Thiosulfate oxidation is the core of this oxidation pathway: first, SoxXA catalyzes the reaction between the sulfane sulfur of thiosulfate and the SoxY-cysteine-sulfhydryl group of the SoxYZ complex, forming a cysteine S-thiosulfonate derivative (SoxYZ-S-S-SO_3_^-^); second, SoxB hydrolyzes sulfate (SO_4_^2-^) from the terminal sulfone (-SO_3_^-^) group of SoxYZ complex; third, Sox(CD)_2_ oxidizes the sulfane sulfur (–S^-^) of the residual SoxY-cysteine persulfide (SoxYZ-S-S^-^) to cysteine-S-sulfate (SoxYZ-S-SO_3_^-^); eventually, the sulfonate moiety (-SO_3_^-^) is hydrolyzed again by SoxB, regenerating SoxYZ (summarized by Ghosh and Dam, 2009; Welte et al., 2009; Zander et al., 2011). However, in contrast to the current Sox metabolic models, a new discovery on the intermediates of the Sox pathway was reported: it was proposed that instead of cysteine S-thiosulfonate (SoxYZ-S-(S)-SO_3_^-^), SoxYZ that conjugates with multiple sulfane atoms (SoxYZ-S-(S)_n_-SO_3_^-^, *n* ≥ 2) is the true carrier species in the Sox pathway (Grabarczyk and Berks, 2017). In addition, Sox(CD)_2_ is absent in a wide range of the Sox-system-dependent sulfur-oxidizing bacteria. In the Sox pathway that lacks Sox(CD)_2_ (so-called truncated Sox pathway), the sulfur atom of the sulfane intermediate (SoxYZ-S-S^-^) is probably fed into other Sox pathways or transformed into storage forms of sulfur (summarized by Ghosh and Dam, 2009; Welte et al., 2009). In recent years, structural and biological studies on Sox proteins, including the analysis of their three-dimensional structures and the identification of active sites, have provided more information regarding the catalytic mechanism of the Sox system at atomic-level resolution (Bamford et al., 2002; Sauve et al., 2007, 2009; Bradley et al., 2012; Grabarczyk et al., 2015).

**FIGURE 5:**
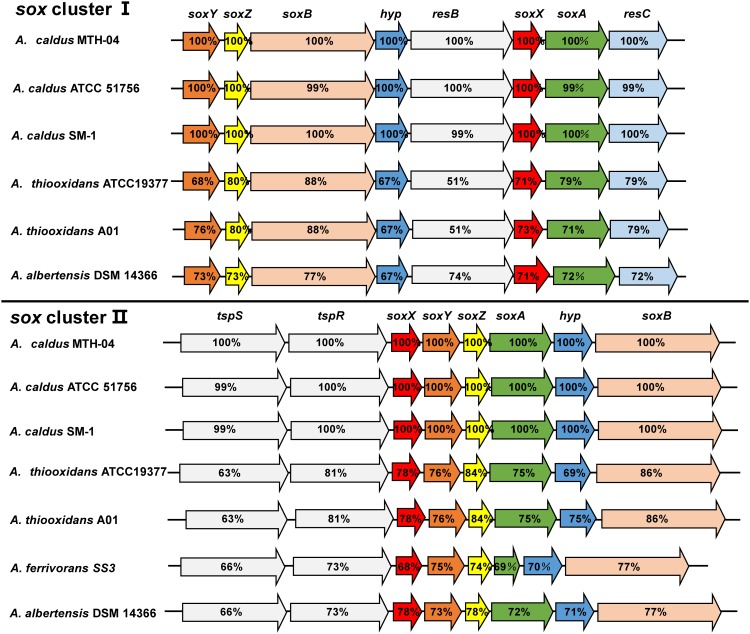
The *sox* clusters in *A. caldus* and *A. thiooxidans*. The percentages of similarities between protein sequences are indicated by the values marked in the gene. *A. caldus* MTH-04: *sox* I (Gene ID: A5904_10510-10475), *sox* II (A5904_11270- 11305); *A. caldus* ATCC 51756: *sox* I (Acaty_c2059- c2052), *sox* II (Acaty_c2206-c2213); *A. caldus* SM-1: *sox* I (Atc_2217-2209), *sox* II (Atc_2363-2370); *A. thiooxidans* ATCC19377: sox I (ATHIO_RS0100375-RS0100340), *sox* II (ATHIO_RS0101665-RS0101630); *A. thiooxidans* A01: *sox* I (X795_RS0118310-RS0118345), *sox* II (RS0104295-RS0104260); *A.albertensis* DSM 14366: *sox* I (BLW97_RS03815-RS03850), *sox* II (BLW97_RS11430-RS11465); *A. ferrivorans* SS3: *sox* II (Acife_2487–2494).

Comparative analysis of the published genomic sequences from species of the genus *Acidithiobacillus* indicated that *sox* clusters without *sox*CD genes are present in *A. caldus*, *A. thiooxidans*, *A. albertensis* and *A. ferrivorans*, but not found in *A. ferrooxidans* strains (Figure 5 and Table 1; Valdés et al., 2008b; Talla et al., 2014; Christel et al., 2016; Castro et al., 2017). Two separate *sox* clusters are present in *A. caldus*, *A. thiooxidans* and *A. albertensis*. One cluster is arranged in the order *soxXYZA-hyp-soxB* that is located downstream of a sigma54-dependent two component system *tspS-tspR* (termed as *sox*-II cluster), the other one was *soxYZB-hyp-resB-soxAX-resC* (termed as *sox*-I cluster). Only the *sox*-II-like cluster was found in *A. ferrivorans* (Figure 5).

Due to the absence of Sox(CD)_2_ protein in the Sox system of *Acidithiobacillus*, two possible ways for SoxYZ regeneration in bacteria with the truncated Sox pathway were proposed, including the cleavage of sulfur atom of the sulfane intermediate (SoxYZ–S–S^-^) and the oxidation of SoxYZ–S–S^-^ by the predicted sulfur dioxygenase (SDO) (Chen et al., 2012; Yin et al., 2014). Since it is hypothetized that SDO is located in cytoplasm where it oxidizes the elemental sulfur to sulfite (Wu et al., 2017), it is inferred that the sulfur atom of the sulfane intermediate (SoxYZ-S-S^-^) should be cleaved to form elemental sulfur via an unknown mechanism, so that SoxYZ is regenerated. As a consequence, studies on SoxYZ regeneration and sulfur globule accumulation are important for uncovering the catalytic process of the truncated Sox system in *Acidithiobacillus* spp.

### Thiosulfate Dehydrogenase (TSD)

The first report on the thiosulfate dehydrogenase in *Acidithiobacillus* was released by Silver and Lundgren (1968b), that the thiosulfate-oxidizing enzyme purified from *A. ferrooxidans* oxidizes one mole of thiosulfate to produce 0.5 mole of tetrathionate. The optimal pH for this thiosulfate-oxidizing enzyme was 5.0 and no cofactor was required for this reaction (Silver and Lundgren, 1968b). Janiczek et al. (2007) described that the purified TSD from *A. ferrooxidans* was a tetramer consisting of four identical subunits of 45 kDa and its optimal activity was observed at pH 3.0. It was not until 2013, that the gene (AFE_0042) encoding thiosulfate dehydrogenase (∼25 kDa) was identified by Kikumoto et al. (2013) Maximum enzyme activity appeared at pH 2.5 and 70°C, but this enzyme could reduce neither ubiquinone nor horse heart cytochrome c. The low pH optimum of TSD indicated that this enzyme probably metabolized thiosulfate in the periplasmic space. It was reported that different TSDs were purified from *A. thiooxidans* (Nakamura et al., 2001), but the corresponding gene sequences are unidentified.

**FIGURE 6:**
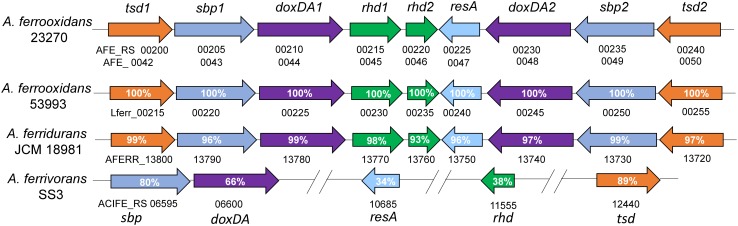
Schematic map of the thiosulfate-metabolic gene clusters in *A. ferrooxidans* and other *Acidithiobacillus* sp. *tsd*, thiosulfate dehydrogenase; *sbp*, sulfate/molybdate binding protein; *doxDA*, thiosulfate:quinol oxidoreductase; *rhd*, rhodanese-like domain-containing protein; *resA*, thiol-disulfide oxidoreductase. The numbers after *tsd, sbp, doxDA* and *rhd* were there to differentiate the two copies of these genes in the cluster. The identities of TSD1 and TSD2, SBP1 and SBP2, DoxDA1 and DoxDA2 in *A. ferrooxidans* are 76, 74, and 71%, respectively.

Using the protein sequence of AFE_0042 in *A. ferrooxidans* as the reference sequence, another potential TSD (AFE_0050) was discovered in *A. ferrooxidans* with 76% identities to the protein sequence of AFE_0042. These two copies of *tsd* genes, together with other thiosulfate-oxidation-related genes (*doxDA* and *rhd*), are arranged in a unique thiosulfate-metabolic gene cluster in the genome of *A. ferrooxidans* (Figure 6). This thiosulfate gene cluster was also discovered in *A. ferridurans*, but does not exist in *A. ferrivorans* that contains TSD (Figure 6). However, no TSD homologs are found in *A. caldus* or *A. thiooxidans* (Table 1). The conflict between the absence of TSD homologs and the reported TSD activity in *A. thiooxidans* should be clarified in further investigations (Nakamura et al., 2001). Taken together, these results suggest that the existence of the TSD-dependent thiosulfate-oxidation pathway is probably a typical character for sulfur- and ferrous-oxidizing bacteria (*A. ferrooxidans, A. ferridurans*, and *A. ferrivorans*).

The most intensively studied tetrathionate-forming thiosulfate dehydrogenase (TsdA) was identified and investigated in the purple sulfur bacterium *A. vinosum* (Denkmann et al., 2012). This enzyme is a periplasmic, monomeric 25.8 kDa c-type cytochrome, most active at pH 4.0. The catalytic mechanism of TsdA has been elucidated (Liu et al., 2013; Brito et al., 2015; Kurth et al., 2016). Homologous proteins are not encoded in *Acidithiobacillus* genomes, indicating potential differences in thiosulfate dehydrogenases (Kikumoto et al., 2013).

The TSD in *Acidithiobacillus* and TsdA in *Allochromatium* can be considered as two different kinds of thiosulfate dehydrogenase based on protein similarity, enzymatic features and their existence in different sulfur-oxidizing bacteria. Thus, investigations on the distribution of TSD and TsdA homologs in different sulfur-oxidizing prokaryotes will be essential for illustrating the differences in thiosulfate metabolism during the evolution of these sulfur-oxidizing species. The unusual gene arrangement of the *tsd-doxDA* clusters in *A. ferrooxidans, A. ferridurans* and *A. ferrivorans* implies a unique thiosulfate-metabolic mode in these sulfur- and ferrous-oxidizing *Acidithiobacillus* spp. Studies on gene function and regulation mechanism of this cluster would provide new insights in thiosulfate metabolism in *A. ferrooxidans*, *A. ferridurans*, and *A. ferrivorans.* Meanwhile, any further knowledge on the protein structure and catalytic mechanism of TSD would be helpful for understanding the TSD-catalyzed thiosulfate-metabolic process in these sulfur-oxidizing bacteria.

## Sulfide Oxidation

Sulfide is an important sulfur-substrate and metabolic-intermediate during elemental sulfur oxidation in *Acidithiobacillus*. The sulfide-oxidizing enzyme SQR is present in different domains of life from prokaryotes to animals (Griesbeck et al., 2000). It has been proved that sulfide can be oxidized to zero-valent sulfur by the membrane-bound protein SQR and at the same time, electrons are generated and fed into the membrane quinone pool (Griesbeck et al., 2000). SQR activity was detected in *A. ferrooxidans* NAsF-1. When cells were cultured in S^0^ medium, the activity was 17 times higher than in Fe^2+^-medium (Wakai et al., 2004). The crystal structure of *A. ferrooxidans* SQR suggested the potential oxidation mechanism: when a sulfide ion interacts with the S^γ^ of Cys356, two electrons are acquired by FAD and the sulfur atom is attached to the polysulfide bridge (Cherney et al., 2010). The active sites of *A. ferrooxidans* SQR includes two cysteines (Cys160, Cys356) involved in the transfer of electrons to FAD and the formation of the polysulfide bridge, a third cysteine (Cys128) related to the release of the polysulfur product, and two histidine residues (His132, His198) that are essential for its function (Cherney et al., 2010; Zhang and Weiner, 2014). Multiple copies of putative *sqr* genes were annotated in the genome of *Acidithiobacillus* strains, but their roles in the sulfur oxidation are still unclear. SQRs have been classified into several groups (Pham et al., 2008; Marcia et al., 2009, 2010; Sousa et al., 2018), and the potential SQRs in a single species of *Acidithiobacillus* may belong to different groups. The different catalytic properties and distinct functions of SQRs in *Acidithiobacillus* spp. could be investigated in future researches to reveal their different roles in the sulfur oxidation of these bacteria.

## Sulfite Oxidation

Sulfite needs to be quickly oxidized or transformed in the organism due to its harmful effect on the cell. Enzyme-mediated sulfite oxidation can occur in the periplasm and the cytoplasm in different bacteria. Besides, sulfite also can be converted to sulfate, thiosulfate or glutathione S-sulfonate chemically with the help of Fe^3+^ or sulfur (Sugio et al., 1987b; Suzuki et al., 1992; Harahuc and Suzuki, 2001).

Sulfite oxidase activity from *A. ferrooxidans* strains was reported (Vestal and Lundgren, 1971; Sugio et al., 1988; Sugio et al., 1992; Suzuki, 1994), but the gene sequence was still unidentified (Valdés et al., 2008a). Until now, the knowledge on sulfite oxidation in *Acidithiobacillus* spp. was mainly acquired based on the prediction of potential genes and inference of protein functions. Three pathways/enzymes are proposed to participate in the sulfite oxidation process in *Acidithiobacillus*: the Sox system that oxidizes the periplasmic sulfite, an adenosine-5’-phosphosulfate (APS) pathway involving two different enzymes that catalyze the cytoplasmic sulfite oxidation and a predicted SoeABC complex.

In fact, similar APS pathways associated with cytoplasmic sulfite oxidation were reported in *A. ferrooxidans*, *A. caldus*, *A. thiooxidans* and *A. ferrivorans* (Quatrini et al., 2009; Chen et al., 2012; Yin et al., 2014; Christel et al., 2016). The APS pathway consists of an APS reductase (AprBA) and an ATP sulfurylase (SAT). These enzymes are involved in the dissimilatory sulfate reduction pathway in sulfate-reducing prokaryotes. SAT utilizes ATP and sulfate to generate APS which is further converted to AMP and sulfite by AprBA. In some phototrophic and chemotrophic sulfur-oxidizing bacteria, this pathway was postulated to work in the reverse direction: sulfite is oxidized to sulfate; meanwhile, substrate level phosphorylation is achieved (Hipp et al., 1997; Meyer and Kuever, 2007; Meyer and Kuever, 2008). The genomic sequence analysis showed that putative *sat* genes encoding enzymes catalyzing turnover of APS to sulfate and ATP are present in *A. ferrooxidans* and *A. caldus*, but AprBA homologs that oxidize sulfite to APS are not encoded in these strains (Quatrini et al., 2009; Chen et al., 2012). In *A. thiooxidans* A01, phosphoadenosine phosphosulfate (PAPS) reductase and adenylylsulfate kinase genes were discovered, while the *sat* gene was not detected (Yin et al., 2014). To elucidate APS-mediated sulfite oxidation process in these sulfur-oxidizing bacteria, more information on the essential genes and their functions is required.

SoeABC, a heterotrimeric membrane-bound complex, was first reported to be involved in sulfite oxidation in the cytoplasm of the chemotrophic *Ruegeria pomeroyi* (Lehmann et al., 2012). The complex is formed by three subunits: an NrfD/PsrC like membrane protein (SoeC), an iron–sulfur protein (SoeB), and a molybdoprotein (SoeA) (Lehmann et al., 2012; Dahl et al., 2013). The membrane-bound iron-sulfur molybdoprotein SoeABC has been identified as a major direct sulfite-oxidizing enzyme in the cytoplasm of the purple sulfur bacterium *A. vinosum* (Dahl et al., 2013). The genes encoding SoeABC were reported in the genome of *A. caldus* SM-1 (Liu L.J. et al., 2014) and SoeABC homologs are also found in *A. ferrivorans*, *A. ferrooxidans*, *A. thiooxidans* and *A. albertensis* (unpublished data), implying the presence of a direct cytoplasmic sulfite-oxidizing pathway in these chemoautotrophic sulfur-oxidizers.

## Sulfur Oxidation Network in *Acidithiobacillus* Spp.

With the identification of new enzymes, application of omics technologies and gene functional studies based on gene knockout techniques, different models of the sulfur-metabolic networks have been proposed to elucidate the sulfur-oxidizing processes in *Acidithiobacillus* species (Hallberg et al., 1996; Valdés et al., 2008a; Mangold et al., 2011; Chen et al., 2012; Bobadilla Fazzini et al., 2013; Yin et al., 2014). Based on the knowledge regarding discovered sulfur-oxidizing enzymes and the previous models, sulfur oxidation in *Acidithiobacillus* spp. can be classified into two modes: one is the Sox-pathway-dependent sulfur oxidation networks in *A. caldus*, *A. thiooxidans*, *A. albertensis*, and *A. ferrivorans*, and the other is the Sox-pathway-independent sulfur-metabolic network in *A. ferrooxidans* (Table 1). The Sox system is present in both the sulfur-oxidizing-only species and the sulfur- and ferrous-oxidizing species *A. ferrivorans*. Results from our laboratory indicate that the Sox-containing strains of *A. caldus* and *A. thiooxidans* have higher sulfur oxidation capacities (higher elemental-sulfur-oxidizing rate and growth density in S^0^-medium) compared with a Sox- deficient *A. ferreooxidans* strain. Although *A. ferrooxidans* has thiosulfate dehydrogenase (TSD), the differences in the abilities of obtaining electrons from TSD and Sox system probably contribute to the different sulfur oxidation capacities of these *Acidithiobacillus* strains. Interestingly, *A. ferrivorans* possesses a single copy of both the *sox* cluster and *tsd* gene while in other species double copies of these genes are found. Based on the important role of periplasmic thiosulfate metabolism in cell growth and the observed differences in thiosulfate-metabolic pathways in *Acidithiobacillus spp.*, it may be suggested that the variations in thiosulfate-metabolic pathways/enzymes should be a key difference of the sulfur oxidation process in different species of *Acidithiobacillus*.

**FIGURE 7:**
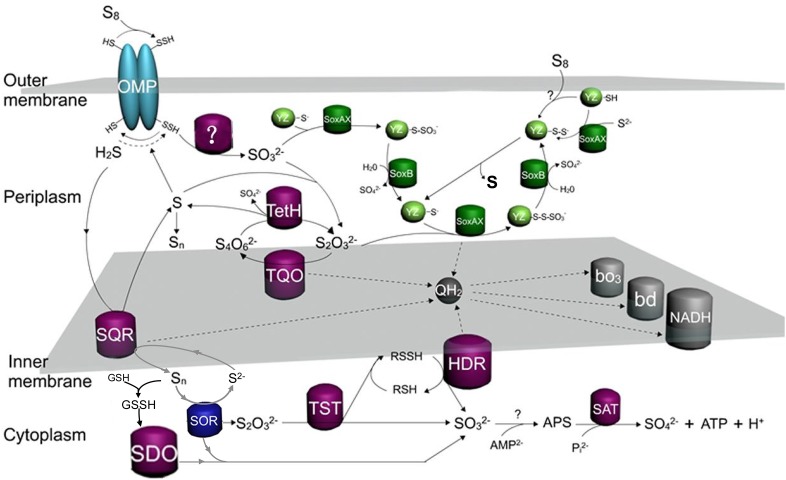
The updated model of sulfur oxidation in *A. caldus*. OMP, outer-membrane proteins; TQO, thiosulfate quinone oxidoreductase; TetH, tetrathionate hydrolase; SQR, sulfide:quinone oxidoreductase; SDO, sulfur dioxygenase; SOR, Sulfur oxygenase reductase; TST, rhodanese; HDR, Hdr-like complex; SAT, ATP sulfurylase; bd, bo_3_, terminal oxidases; QH2, quinol pool; NADH, NADH dehydrogenase complex I.

**FIGURE 8:**
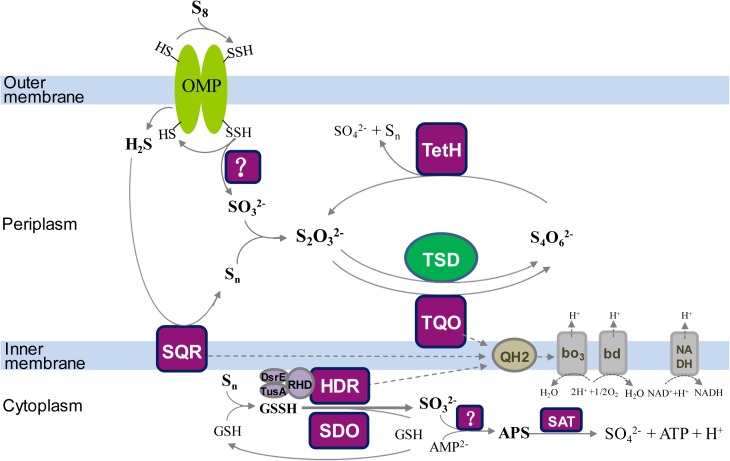
The updated model of sulfur oxidation in *A. ferrooxidans*. OMP, outer-membrane proteins; TQO, thiosulfate quinone oxidoreductase; TSD, thiosulfate dehydrogenase; TetH, tetrathionate hydrolase; SQR, sulfide:quinone oxidoreductase; SDO, sulfur dioxygenase; HDR, Hdr-like complex; SAT, ATP sulfurylase; bd, bo_3_, terminal oxidases; QH2, quinol pool; NADH, NADH dehydrogenase complex I.

To better understand the Sox-pathway-dependent and Sox-pathway-independent sulfur-metabolic processes in *Acidithiobacillus*, modified sulfur oxidation models for *A. caldus* and *A. ferrooxidans* were proposed based on cutting-edge research progresses (Valdés et al., 2008a; Quatrini et al., 2009; Mangold et al., 2011; Chen et al., 2012). In *A. caldus* (Figure 7), extracellular elemental sulfur (S_8_) is activated and transported by special outer-membrane proteins (OMP) into the periplasm where persulfide sulfane sulfur is oxidized by an unknown enzyme; the resulting sulfite can directly enter the Sox pathway or form S_2_O_3_^2-^ via a non-enzymatic reaction between SO_3_^2-^ and a sulfur atom; the periplasmic thiosulfate is then processed by the truncated Sox pathway, producing sulfate and elemental sulfur, or be catalyzed by TQO to generate S_4_O_6_^2-^ that is further hydrolyzed by TetH; the hydrogen sulfide generated in the activation of S_8_ is oxidized by SQR located in the inner membrane; some sulfur-metabolic processes, including the truncated Sox pathway mediated thiosulfate metabolism, tetrathionate hydrolysis and sulfide oxidation, can produce elemental sulfur that may be re-activated and -oxidized at the outer membrane region, or be mobilized into the cytoplasm where it is oxidized by SDO and SOR; the products formed by SDO and SOR can activate the cytoplasmic sulfur-metabolic pathways including the pathway of S_2_O_3_^2-^ utilization catalyzed by rhodanese (TST) and the Hdr-like complex (HDR), the degradation of SO_3_^2-^ via the APS pathway and the oxidation of S^2-^ by SQR. Sulfur oxidation in *A. ferrooxidans* differs from that of *A. caldus* in its absence of the Sox pathway, however, it possesses TSD that could be an alternative thiosulfate-metabolic pathway in the periplasm (Figure 8).

The discovery of a TSD homolog in Sox-system-containing *A. ferrivorans* indicates the existence of a hybrid thiosulfate-metabolic mode in the periplasm of this species of *Acidithiobacillus*. Some of the sulfur-oxidizing pathways/enzymes, including TQO in the S_4_I pathway and the Hdr-like complex and SQR, are proposed to feed electrons via the quinol pool (QH_2_) in the inner membrane to the terminal oxidases *bd* or *bo*_3_ for producing ATP or to NADH dehydrogenase (complex I) for generating NADH (Figures 7, 8; Valdés et al., 2008a; Quatrini et al., 2009; Mangold et al., 2011; Chen et al., 2012; Yin et al., 2014; Christel et al., 2016).

The sulfur-metabolic networks in *Acidithiobacillus* spp. differ from those of the sulfur-oxidizing archaea (Kletzin et al., 2004; Liu et al., 2012). Regarding the thiosulfate metabolism, the S_4_I pathway exists in sulfur-oxidizing archaea such as *Acidianus ambivalens* (Muller et al., 2004; Wang et al., 2016), but there is no report on the Sox system and thiosulfate dehydrogenase (TSD) in these archaeal sulfur-oxidizers. At least two of the three thiosulfate metabolic pathways/enzymes (Sox system, S_4_I pathway and TSD enzyme) are employed in each species of *Acidithiobacillus*. The complexity of thiosulfate metabolism could be considered as a difference between *Acidithiobacillus* and sulfur-oxidizing archaea. As for elemental sulfur oxidation, SOR plays a critical role in sulfur-oxidizing archaea (Kletzin et al., 2004), but it is not an indispensable enzyme in *Acidithiobacillus* spp. (You et al., 2011; Chen et al., 2012). In these chemoautotrophic sulfur-oxidizing bacteria, SDO is one of the important cytoplasmic elemental-sulfur-oxidizing enzymes (Figure 1; Wang et al., 2014; Wu et al., 2017). Meanwhile, there are sulfur-oxidizing genes possessed by both sulfur-oxidizing archaea and *Acidithiobacillus*, such as *dsrE-tusA*, *soeABC*, and *hdrC1B1A-hyp-hdrC2B2* genes (Liu L.J. et al., 2014).

Sulfur oxidation in *Acidithiobacillus* spp. is a sophisticated process that involves various enzymes/proteins and sulfur compounds in different cellular compartments. The proposal of sulfur oxidation models provides an overall and systematic understanding of the sulfur-metabolic process in these bacteria. However, there are still some questions and doubts regarding these models: (i) The localization of these sulfur-oxidizing enzymes in the cell. For example, SQR is proposed to be located at the periplasmic surface of the cytoplasmic membrane in the published models of *Acidithiobacillus* spp. (Valdés et al., 2008a; Quatrini et al., 2009; Mangold et al., 2011; Chen et al., 2012; Yin et al., 2014; Christel et al., 2016). Similar localization of SQR was reported for *Rhodobacter capsulatus* (Schutz et al., 1999), while results for heterotrophic *Cupriavidus pinatubonensis* indicated that SQR located on the cytoplasmic side of the membrane and the soluble cytoplasmic PDO was in the vicinity of the membrane (Gao et al., 2017). The close location of SQR and PDO in the cytoplasm increases the efficiency of sulfide oxidation in heterotrophic bacteria (Gao et al., 2017). However, whether the SQR in *Acidithiobacillus* spp. works in the same way as that of heterotrophic bacteria remains unknown. (ii) The actual sulfur-intermediates in the metabolic process. Various reduced sulfur compounds are produced during the oxidation of elemental sulfur to the final product sulfate, and some of them (thiosulfate, tetrathionate, sulfane sulfur and so on) are hypothesized to be important sulfur-substrates for sulfur-oxidizing pathways in *Acidithiobacillus* spp. models (Valdés et al., 2008a; Quatrini et al., 2009; Mangold et al., 2011; Chen et al., 2012; Yin et al., 2014; Christel et al., 2016). However, experimental confirmation on the existence of these sulfur-intermediates *in vivo* is lacking, thus the actual intracellular transformation of sulfur compounds is obscure. (iii) Electron transport chain. The quinol pool was proposed to mediate the electron transport in *Acidithiobacillus* spp. In published models, it was proposed that the electrons generated by the Sox complex were transferred via the quinol pool (Valdés et al., 2008a; Quatrini et al., 2009; Mangold et al., 2011; Chen et al., 2012; Yin et al., 2014; Christel et al., 2016). However, because of the presence of cytochrome c (SoxXA) in the Sox system (Mangold et al., 2011; Chen et al., 2012; Yin et al., 2014; Christel et al., 2016), the electrons obtained from Sox complex could be transferred directly to cytochrome c oxidase via SoxXA. Thus, which is the actual electron transport for the Sox system in *Acidithiobacillus* spp. remains to be verified.

## Regulation of Thiosulfate-Metabolic Pathways in *Acidithiobacillus* Spp.

How to sense the sulfur-metabolites and regulate their sulfur-metabolic pathways is fundamental for these sulfur-oxidizers to catalyze various RISCs. The TCS, including a membrane-bound sensor histidine kinase HK and a cognate response regulator RR, is a predominant regulatory mechanism for prokaryotic microorganisms to initiate specific adaptive responses in response to environmental stimuli (Capra and Laub, 2012). *Acidithiobacillus* spp. have evolved a set of TCSs to regulate the thiosulfate metabolism in the periplasm. TCS genes upstream of the *tetH* gene of S_4_I pathway were found in *A. caldus*, *A. ferrooxidans* and *A. ferridurans*, but not in other species of *Acidithiobacillus* (Rzhepishevska et al., 2007; Wang et al., 2016). Phylogenetic analysis indicated that RsrS/RsrR in *A. caldus* was an EnvZ/OmpR-like TCS, whereas the TcsS/TcsR in *A. ferrooxidans* and *A. ferridurans* was a σ^54^-denpendent TCS similar to ZraS/ZraR-like systems (Rzhepishevska et al., 2007; Wang et al., 2016). EnvZ/OmpR mediates osmotic stress response in various gram-negative bacteria, and ZraS/ZraR (HydH/HydG) is found responding to high concentrations of zinc or lead in the medium (Leonhartsberger et al., 2001; Cai and Inouye, 2002). The TspS/TspR that regulate the Sox pathway was also a σ^54^-dependent TCS, located upstream of *sox*-II cluster in both *A. caldus* and *A. thiooxidans* (Li et al., 2017). In *A. ferrooxidans*, the global redox responding TCS RegB/RegA could regulate the ferrous iron and RISC oxidation pathways (Sandoval Ponce et al., 2012; Moinier et al., 2017).

**FIGURE 9:**
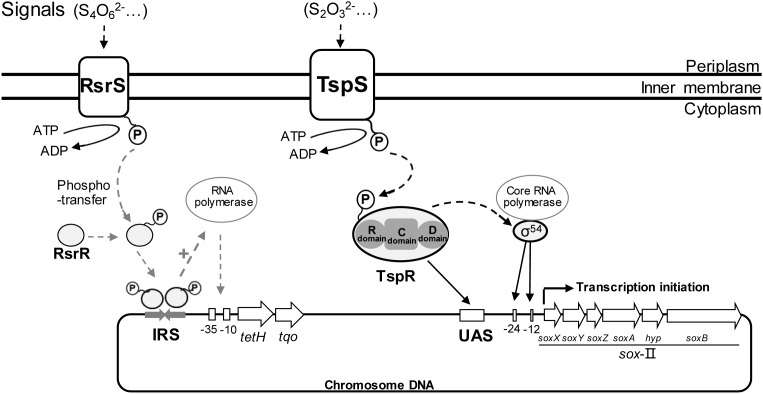
Regulation model of periplasmic thiosulfate metabolism in *A. caldus*. RsrS/RsrR and TspS/TspR regulates the transcription of *tet*H and *sox*-II gene cluster, respectively.

Based on previous results on RsrS/RsrR and TspS/TspR (Wang et al., 2016; Li et al., 2017), a new regulation model (shown in Figure 9) is summarized to better illustrate the significance of TCSs in the regulation of the periplasmic thiosulfate-metabolism in *A. caldus*. In the model, S_2_O_3_^2-^, the potential signal molecule, is sensed by TspS and the activated TspS transmits the signal to TspR; the phosphorylated TspR binds to the upstream activator sequence (UAS; TGTCCCAAATGGGACA), initiating the transcription of *sox*-II gene cluster to express the Sox system for the metabolism of thiosulfate; S_2_O_3_^2-^ can also be oxidized by TQO to generate S_4_O_6_^2-^ that is later sensed by RsrS; the signal is transferred to RsrR, generating the active dimer. The RsrR dimer combines to a 19bp-inverted-repeat-sequence (IRS, AACACCTGTTACACCTGTT), stimulating the transcription of *tetH* and *tqo*, thus tetrathionate is decomposed to regenerate thiosulfate. Therefore, both the regulation of TspS/TspR of the Sox system and RsrS/RsrR of the S_4_I pathway are important for maintaining the balance between the oxidation and the conversion of S_2_O_3_^2-^. The existence of TCSs that are associated with thiosulfate metabolism regulation in the periplasm of *A. caldus* and other acidophilic sulfur-oxidizers, allows these sulfur-oxidizing microbes to detect the generated S_2_O_3_^2-^ in the periplasm, and then modulate thiosulfate-metabolic pathways to quickly oxidize the instable S_2_O_3_^2-^ or convert it to the acid-stable S_4_O_6_^2-^. The discovery of the regulation of TCSs on the S_4_I and Sox pathways also indicates the influence of these thiosulfate-metabolic pathways on the sulfur-metabolic network of *Acidithiobacillus* spp.

## Conclusion and Future Directions

Sulfur oxidation in chemoautotrophic *Acidithiobacillus* is an important component of microbial sulfur metabolism in the global sulfur cycle. Research on sulfur oxidation of *Acidithiobacillus* has made remarkable progresses in multiple aspects over the past decades, from enzymological studies and gene identification to protein structure and catalytic mechanism. The establishment of sulfur oxidation models for *Acidithiobacillus* spp. provides overall understanding of the sulfur-metabolic process. Sulfur metabolism in *Acidithiobacillus* spp. involves various sulfur-oxidizing pathways and enzymes located in different compartments of the cell, indicating the complexity and diversity of sulfur oxidation in these acidophilic autotrophic bacteria. Gene regulation systems including TCSs and other undetected regulatory mechanisms, also contribute to the remarkable sulfur-oxidizing abilities of *Acidithiobacillus* spp. However, detailed and in-depth studies on gene function and enzymatic properties involved in sulfur oxidation are insufficient or absent, resulting in the ambiguous conclusions or questions without answers in certain aspects of sulfur metabolism in *Acidithiobacillus*. (i) The reason for the existence of multiple copies of sulfur-oxidizing genes (*rhd*, *dsrE*, *sox* and so on) and their different roles in sulfur oxidation; (ii) the enzymatic properties of some proteins in *Acidithiobacillus* are speculated but not experimentally confirmed, such as the Hdr-like complex, SoeABC and the APS-sulfite-oxidizing pathway (Quatrini et al., 2009; Liu L.J. et al., 2014); (iii) structural analysis and catalytic mechanism of some sulfur-oxidizing enzymes (TSD, TetH, and TQO) remains to be clarified. Moreover, the utilization of various omics technologies at DNA, RNA and protein levels to study these sulfur-metabolic genes would facilitate the discovery of new sulfur-oxidizing proteins and improve the understanding of the sulfur-metabolic networks in *Acidithiobacillus* strains. Besides, the developments of novel methods or techniques, such as methods for visualization of protein localization and sulfur-metabolite detection techniques *in vivo* and *in vitro*, are needed to facilitate the studies of the enzymatic function and catalytic process in *Acidithiobacillus* spp. and other sulfur-oxidizing microorganisms.

## Author Contributions

RW, J-QuL, and L-XC designed and composed the manuscript. J-QiL, X-ML, and XP helped in revising the manuscript. C-JZ, X-YG, C-ML, Y-QL, and YL prepared the figures. C-LY analyzed the gene and protein sequences.

## Conflict of Interest Statement

The authors declare that the research was conducted in the absence of any commercial or financial relationships that could be construed as a potential conflict of interest.
